# How COVID-19 affects the use of evidence informed policymaking among iranian health policymakers and managers

**DOI:** 10.1186/s13690-021-00757-3

**Published:** 2022-01-05

**Authors:** Peivand Bastani, Jamshid Bahmaei, Ebrahim Kharazinejad, Mahnaz Samadbeik, Zhanming Liang, Carmen Huckel Schneider

**Affiliations:** 1 Health Human Resources Research Center, School of Management and Medical Informatics, Shiraz University of Medical Sciences, Shiraz, Iran; 2 Student Research Committee, Shiraz University of Medical Sciences, Shiraz, Iran; 3 Department of Anatomical Sciences, Abadan University of Medical Sciences, Abadan, Iran; 4 Social Determinants of Health Research Center, Lorestan University of Medical Sciences, Khorramabad, Iran; 5 Centre for Online Health, Faculty of Medicine, The University of Queensland, Brisbane, Australia; 6 College of Public Health, Medical and Veterinary Sciences, Division of Tropic Health and Medicine, James Cook University, 4811 Townsville, Qld Australia; 7 Menzies Centre for Health Policy and Economics, Sydney School of Public Health, University of Sydney, Sydney, Australia

**Keywords:** Evidence informed Policy making, Knowledge Translation Exchange, Knowledge Broker, COVID-19, Health policymakers, Health managers, Iran

## Abstract

**Background:**

The COVID-19 pandemic increased the need for new valid scientific evidence to support urgent clinical and policy decision making; as well as improved processes for the rapid synthesis, uptake and application of that evidence. Evidence informed policymaking (EIPM) can be considered as a way to access and use the results of evidence in practice. This study aimed to determine what effects COVID-19 had on the way Iranian health managers and policymakers use evidence in their decisions.

**Methods:**

This study was conducted in 2021 applying a qualitative research design. Data was collected through semi-structured interviews. Thirty health care managers, policy makers and medical university faculty members were recruited as the study participants, initially via a purposive sample, followed by snowballing. A conventional content analysis presented by Hsieh and Shannon (2005) was applied for data analysis.

**Results:**

Ten main themes emerged from the data including: 1) roles and duties of knowledge brokers (KBs); 2-5) the roles, benefits, barriers and necessities of applying Knowledge Translation Exchange (KTE) tools; 6-8) the facilitators, benefits and barriers to the application of evidence during COVID-19; 9) challenges of rapid evidence production evidence during COVID-19 and 10) consequences of not applying evidence during COVID-19. According to the present conceptual framework, KBs act as an intermediator between the large amounts of knowledge produced and decision makers. KTE tools should be applied to enhance EIPM during COVID-19. Attention should be paid to the facilitators, barriers, benefits and necessities of evidence application during COVID-19 to avoid negative consequences for the health system.

**Conclusions:**

Results of this study show that developing KTE tools and activating KBs can be among the main strategies to produce applied actionable messages for policymakers to move toward EIPM; and that this applies even when rapid decision making is required, such as during the COVID-19 pandemic. It is strongly recommended to reinforce the local capacities through supporting scientific networks and relationships between research centers and local and national policymakers. At the same time, attention to local barriers to and facilitators of the application of evidence while facing a pandemic can pave the way to better identification of health system`s problems and rapid responses.

## Background

Corona Virus Disease 19 (COVID-19) has been the worst public health disaster in recent decades and it has severely impacted on health systems and economies all over the world. Such an emergency has placed unexpected pressures on decision makers and policymakers at all the levels to make urgent decisions with wide ranging consequences. These decisions have had to be made despite high levels of uncertainty and significant long term consequences for the lives of millions of people [1].

As COVID-19 spread rapidly throughout the world, managers and policymakers needed to be ready for a rapid response. They required both timely and relevant evidence to determine the most promising, implementable, and appropriate courses of action to prevent and control disease. During the COVID-19 pandemic, the speed of production and dissemination of information was rapid, including clinically relevant evidence (e.g. diagnostics, treatments, personal protective equipment, vaccines and other technologies) as well as public health evidence (e.g. severity of disease, infectiousness, models of likely spread) and health protection evidence (e.g. the effectiveness of social distancing, curfews, and school closures). As a result, health care managers, decision makers and policymakers had to deal with very large amounts of trusted and untrusted information; posing a challenge for established decision making processes [2].

In such large scale disasters, the appropriate understanding, interpretation, use and translation of evidence is even more important as it can have lifesaving consequences [2].

However, despite the large amount relevant evidence that has emerged there have been several known instances of policy decisions that have not aligned with the evidence base. For example, in spite of evidence emphasis on the significance of travel ban, social distancing and quarantine, some underdeveloped and developing countries could not implement them thoroughly and effectively.

Research into evidence use over the past 15 years has found that several factors can influence policy makers to take decisions that are contrary to the best available evidence, including political powers, stakeholders` pressures and the economic factors [3].Time pressures created by the urgency of the pandemic response also put pressure on health managers and policymakers to prioritize fast decisions over a slower process that requires pouring over large amounts of evidence or waiting for better evidence [1].

That body of research has also shown that policymakers have always encountered many barriers to EIPM; including a lack of available timely evidence, insufficient skills in retrieving and interpreting evidence and lack of access to evidence (including relationships with the producers of evidence). The established literature on EIPM offers some insights into how EIPM can be increased [4], such as are having access to research publications; having summaries and synthesis of policy relevant evidence, including policy briefs; [5] upskilling decision makers to be able to understand and interpret scientific evidence; increasing the level interaction between researchers and policymakers; and having supportive and organizational culture that value the use of evidence [6, 7]. The literature has also emphasized the need for developing a stronger bridge between research bodies and policymakers through organizing Knowledge Brokers (KBs)[8]. Such brokers can act as an axis of government for increasing the capacity of public policymaking [9]. KBs can facilitate Knowledge Translation and Exchange (KTE) and play important role in providing timely evidence. KBs have been shown to be a popular KTE strategy to improve mutual interaction between the researchers and policymakers as the end users of the knowledge and promote the capacity for EIPM [1, 10–12].

A question remains however as to the extent to which these facilitators also apply during emergencies such as the Covid-19 pandemic, where research and publication timeframes mismatched with the time pressures on decision making. There was also a much higher reliance on data from daily surveillance, scenario modelling and cross jurisdictional policy learning, than in the more commonly depicted ‘pipeline’ models of evidence production, publication, and transfer.

In Iran, policymakers have faced many problems in pandemic management because of barriers to EIPM; including decision makers` negative beliefs toward EIPM, cultural and organizational barriers, lack of trust in local evidence and a serious gap among the national research centers and the health policymakers. In another words, despite a high volume of health research centers in the country that are funded by Ministry of Health and Medical Education (MOHME), most of the research findings have not been used in governmental decision making. To the best of our knowledge, a qualitative study exploring the nature of evidence utilization during the pandemic, particularly among developing countries which may have the similar settings has not been conducted. This study was therefore conducted to determine how COVID-19 has affected the use of evidence by Iranian managers and policymakers.

## Methods

### Study setting

This study was conducted during June-August 2021 applying a qualitative research design. The study settings consist of the Iranian universities of medical sciences affiliated with MOHME. The structure of the Iranian health system includes MOHME and public universities that are responsible for population health and local policymaking. All hospitals, health centers, research centers and faculties of health and medicine are administrated under the supervision of these universities.

### Study population

The study population consisted of Iranian policymakers and managers in healthcare sector with at least three years of scientific or executive experience. In total, 30 of them were recruited via snowball sampling. 5 of them were selected purposefully, then, they were asked to introduce the study team to other experts who could participate. Demographic characteristics of the participants including age, marital status, education level, specialty and managerial experience were also registered. The interviews were continued until saturation level where no new themes were explored.

### Data collection

In depth interviews were applied to gather comprehensive insights from the experts. One researcher (JB) collected the data during April and May 2021.

A literature review and the expert opinions of scholars in the areas of health policymaking and health policy informed the development of the draft of the interview guide. Then, in order to assure the meaningfulness and validity of the questions, pilot interviews were conducted with two faculty members in the area of health policy. Some corrections were done to finalize the interview guide. The final interview guide consisted of a warm up question, some main and sub-questions and probing questions. As a warm up question we asked the participants whether they have used the evidence in their decisions and if they think the pandemic may change this condition.

The interview sessions were held following informal prior coordination with the participant in a time and place preferred by the participants outside of regular work environments. All participants were given full information about the study before obtaining consent to participate. The duration of the interviews varied from 40 to 55 min. All the interview sessions were recorded by two electronic devices with the participants` permission. For more accuracy, any non-verbal gestures of the participants were noted during each session. As the interviews were in Persian, the quotes were translated from the original by members of the study team (indicated by initials in this article). Team members are experienced researchers and published journal articles extensively in English.

### Data analysis

The conventional content analysis presented by Hsieh and Shannon (2005) was applied for data analysis [13]. Conventional content analysis is suggested when the existing theory or research literature about a phenomenon is restricted. The main nature of this approach is applying open coding from the collected data and preventing preconceived categories. In this regard, for analyzing the data in this study, data analysis commenced simultaneous with the data collection process. Right after each interview session, all the recorded contents were listened to several times and converted verbatim to transcripts. Then the initial meaningful units were highlighted after several revision and reading of the whole text. At the same time, a version of transcription was mailed to the interviewees to assure the content of the texts as a process of member check. After repeated readings of the whole text, the meaningful units were highlighted and the initial codes were merged and labeled. Then, through categorizing and refining these codes, the final codes were defined. Next, after combining and reorganizing the final codes, the sub-themes were formed and finally the main themes defined by categorizing the sub-themes. At this step the definition for each theme and sub-theme were organized in table format. For better illustration of each concept, a conceptual framework was developed. MAX QDA software version 10 was applied for data management and data analysis.

### Trustworthiness criteria of the qualitative study

To assure the credibility and trustworthiness of the data, four criteria proposed by Lincoln and Guba were applied: credibility, transferability, dependability and confirmability [14].Long-term participation and interaction between the researchers and the participants assured credibility. A step by step repetition in the process of collection and analysis of the data and also the conditions of the informants were applied to assure dependability. For achieving transferability of the data, a deep description of the data was prepared accompanied by determined process of coding and analysis of the gestures and texts. And, finally, to gain confirmability, cross check with other members of the study team was applied for assuring accuracy of the data along with peer check and expert check. For this purpose, the coding and extracting categories were revised by qualitative experts without any conflicts of interest.

### Ethical considerations

This study is approved by Abadan University of Medical Sciences Ethics Committee with the approval number of IR.ABADANUMS.REC.1399.19.9.

## Results

Thirty experienced healthcare system managers and policy makers participated in the interviews. Table 1 details their characteristics.

**Table 1:** Characteristics of the health managers and policymakers participated in the study

Variable	Response category	N/(%)
**Sex**	Male	12(40)
	Female	18(60)
**Marital status**	Married	30(100)
**Managerial experience (years)**	5-10	3(10)
	10-15	7(23)
	15-20	9(30)
	20-25	3(10)
	25-30	8(27)
**Faculty member**	Yes	23(76)
	No	7(24)
**Area of policy making**	Education	9(30)
	Health	5(17)
	Research	4(13)
	Treatment	7(23)
	Administration and finance	5(17)

*N stands for number of the participants

After analyzing the content of these 30 interviews, 10 main themes and 95 sub-themes were identified. The main themes were: (1) roles and duties of KBs during COVID-19, (2) roles of KTE in COVID-19, (3) the benefits of applying KTE in COVID-19, (4) the barriers for applying KTE in COVID-19, (5) the necessities of applying KTE during COVID-19, (6) the facilitators of applying evidence during COVID-19, (7) the barriers for applying evidence during COVID-19, (8) the benefits of applying evidence during COVID-19, (9) the necessities of producing evidence during COVID-19, and, (10) the consequences of not using evidence during COVID-19 (Table A-Supplement).

### Roles and duties of knowledge brokers during COVID-19

Knowledge Brokers (KBs) were considered by the participants as the intermediate organizations that play a mutual and interactive role between the research centers as the main knowledge producers and policymakers and the managers as the end users of that knowledge. Such organizations can easily create an applied context for transmitting the users` information and knowledge needs to the researchers and help them translating their research outcomes. One participant declares that:

*“During the pandemic, with its unknown nature, the priority of decisions was really important. In such a context, a lot of evidence was presented that could be applied for better policy making. If we could move toward defining and applying knowledge brokers, they would have helped us faster and more effective access to the research results and better prioritizations of the policies…” [P*_*2*_ *].*

### Roles of KTE during COVID-19

Knowledge exchange translation (KTE) is a concept that implies a systematic and dynamic process.

for the application of knowledge in order to improve a community`s health, provide more effective services and strengthen the healthcare systems. In another words KTE acts as a bridge between the researchers and the policymakers and managers to transfer the actionable messages extracted from the researches for better decision making. At the same time KTE can help synthesizing and building recommendations from the available researches as a base for the policymakers and managers` decisions and best practices. About the role and importance of KTE one of the participants added:

*“KTE can be applied as a tool during the pandemic to translate the scientific and complex concepts to the actionable messages that can be used by the managers and policymakers. For instance, it was an obligation for each of the universities of medical sciences to produce a policy note applying the results of their research and the valid evidence and share it with the others. This can be help a lot”[P*_*5*_ *].*

### Benefits of applying KTE during COVID-19

About the benefits of KTE the present participants emphasized that:

*“Applying practical evidence during the pandemic can easily lead to better clinical decision making and more appropriate policies. Also it can help to increase the health literacy of the community in the area of vaccines and other preventive areas against the pandemic” [P*_*7*_ *].*

Or elsewhere another participant stated:

*“KTE led to implementation of the policies that resulted in public behavioral changes and also the changes in the people`s lifestyle like social distancing, wearing facemasks, staying home and so on” [P*_*18*_ *].*

### Barriers of applying KTE during COVID-19

It is important to mention that applying KTE in a developing country encounters many cultural, structural and executive barriers. In this regard, one of the participants describes the barriers as follows:

*“One of the main barriers in implementing KTE in our country is unfortunately overcoming preferences and the political benefits towards EIPM. Such a barrier can widely affect the appropriate process of decision making and policy making” [P*_*10*_ *].*

Another participant added:

*“there are many published articles in the area of COVID-19 but unfortunately we don’t have access to all of them. Another barrier I think is related to this problem that many of our managers and even policy makers are not familiar with the concept of KTE in practice” [P*_*12*_ *].*

### Necessities of applying KTE during COVID-19

The participants mentioned that KTE is in itself is a useful concept, but it was more important to consider the practical tools through which it is operationalized.

*“I don’t it is possible to engage in KTE unless the valid, timeliness and accurate knowledge is available and accessible. Without these pre-requisites applying the present results may be more harmful” [P30].*

Another participant indicated that:

*“Without production the actionable messages according to the stakeholder`s wants and tendencies, KTE can`t be useful. Our community is not research literate enough to understand the results of many scientific research unless it can be translated according to their knowledge and level of literacy” [P*_*14*_ *].*

### Facilitators of applying the evidence during COVID-19

Participants also considered the facilitators to implementing EIPM according to the community`s acceptance and needs. One participant said:

*“We can say one facilitator here has the right beliefs and positive attitudes of the health managers and policymakers regarding the application of evidence in decision making. Their training also can be helpful” [P*_*16*_ *].*

Another participant added:

*“in my opinion, the culture and organization atmosphere toward the research and applying the results can be as a facilitator. Another important factor is whether the manager or the policymaker who is expected to make decisions was appointed according to merit and having scientific understanding and skills or not” [P*_*27*_ *].*

And similarly, another participant pointed to the process of retrieving the evidence as a facilitator.

*“it is really important that policymakers have the possibility and opportunity of timely access to the evidence before their final decisions. A policymaker normally doesn’t have time for searching and retrieving the evidence…” [P*_*33*_ *].*

### Barriers of applying evidence during COVID-19

There are different barriers to applying evidence in policymaking during pandemic. Among them are organizational, legal, cultural, structural and financial barriers. A participant implied that:

*“many of the barriers are related to the organizations. For instance, a policymaker that always acts traditionally with no creativity, can`t use evidence in his/her decision making. He is not even aware of such a thing and is just occupied with his routine and daily duties; with no experience of applying evidence in policymaking…” [P*_*19*_ *].*

Another participant said:

*“sometimes the policymakers want to use scientific evidence in their decisions but the regulations restrict them. The bureaucratic structure of the organization, especially in the governmental sector, can make it much worse” [P*_*20*_ *].*

### Benefits of applying evidence during COVID-19

The participants also pointed to many benefits of EIPM during the pandemic. For example, one participant clarified:

*“the experience of the pandemic shows us how applying the scientific evidence in our decision making can help us avoid trial and error, duplicated actions and waste of time and resources” [P*_*22*_ *].*

Another participant also added:

*“applying evidence helps us know the nature of the disease better and faster particularly regarding different conditions of the regions or different environments like hospitals, health centers, laboratories, …” [P*_*29*_ *].*

### Production of evidence production during COVID-19

According to the participants it is important to pay attention to how evidence is produced and applied during the pandemic. One participant stated that:

*“one necessity is that during pandemic, along with producing the evidence, we should pay attention to the quality of the produced evidence” [P*_*25*_ *].*

Another participant indicated that:

*“ethical considerations are among the most requirements of producing the evidence during pandemic. It is important not to underestimate the problems of vulnerable groups such as elderly population. It is also significant for producing and applying the evidence related to vaccines” [P*_*35*_ *].*

### Consequences of not applying evidence during COVID-19

According to the participants` viewpoints, lack of attention and neglecting the use of evidence in policymaking during pandemic may lead to negative consequences. For instance:

*“a negative consequence can be a situation that the health organization cannot move forward and the trial and error approach will be used. This can threaten the organization`s efficiency and effectiveness” [P*_*31*_ *].*

Another participant added:

*“those policymakers who ignore the evidence during the pandemic, may lose a great deal of time and resources; human resource, financial and the equipment. All these can lead to trial and error, organization collapse and conflict as well as frustration and dissatisfaction” [P*_*17*_ *].*

And finally another participant stated:

*“another consequence of not using scientific evidence in the process of policymaking is that the community will experience the frustration and exhaustion; and mental problems will increase. Such a community may face a delay in returning to normal conditions” [P30].*

**Fig. 1:**
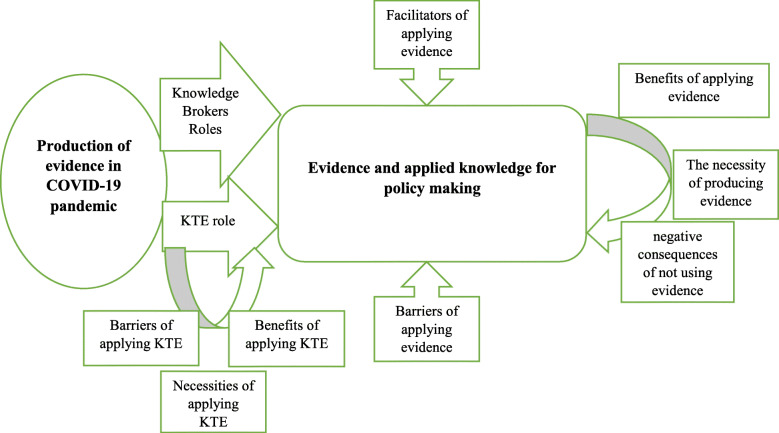
The conceptual model for applying scientific produced evidence by policymakers during COVID-19

Figure 1 illustrates the relationship among these 10 themes via a conceptual model. In the model, the uptake of results of scientific research during COVID-19 pandemic would be increase with the use of KBs and tools of KTE. In another words, through an appropriate process of KTE, an actionable message can be extracted from the relevant evidence according to the audiences and end users` needs and KBs can play an active and interactive role as an intermediate chain in connecting the research centers and the end users. Applying scientific evidence by the policymakers through KTE has accompanied by some barriers like cultural, organizational and structural barriers along with financial barriers to access the evidence. While these barriers could be overcome, some benefits would be achieved for health policymakers and health systems. The necessity of transferring and disseminating the results of the evidence during COVID-19 pandemic.

## Discussion

The aim of this study was to determine how policymakers apply scientific evidence to guide related decisions during COVID-19 pandemic. Our conceptual model reflects the themes that emerged among health care managers and policy makers (Fig. 1) demonstrating the different factors that can affect evidence utilization during the COVID-19 pandemic.

According to this conceptual model, using Knowledge Brokers and KTE tools such as actionable messages, policy briefs, dialogues, and implications for policy makers can facilitate the process of EIPM. Our findings align with Dobbins et al. (2009) who also found that KBs could help build a deeper understanding between the needs of both evidence producers and end users. At the same time, KBs can facilitate interaction and sharing the information among the producers and users of the evidence [15].

Kuchenmüller et al. (2021) have also emphasized that global policy organizations such as the World Health Organisation (WHO) have supported scientific evidence for empowering and training policymakers during COVID-19 pandemic. They have also suggested that WHO has played a significant role as an organized knowledge broker globally [16].

There have however been some use of KTE tools such as policy briefs, which were issued by The World Health Organization (WHO) with some recommendations for evidence based recommendation on appropriate responses to COVID-19 via breaking transmission chains, diagnosis and treatment of the disease and reinforcement of health systems[17].These policy briefs should introduce evidence from systematic reviews at the top of evidence pyramid to guide providing policy options. Such evidence can clearly determine the impacts of policy interventions and pave the way for better understanding along with benefits and disadvantages of every intervention [18, 19]. Figueras et al. (2020) have also stated that during COVID-19, policymakers would benefit from the role of valid, reliable and fair KBs in order to apply evidence in decision making[20]. For the Iranian context and those settings with the similar conditions, it is recommended to improve knowledge and understanding of the concept of KTE among the policymakers and health managers and engage knowledge brokers.

According to the present results, KTE is considered as another concept and process, during which tools can be applied to facilitate EIPM during COVID-19 pandemic. El-Jardali et al. (2020) have also declared that a purposive approach to KTE is helpful in situations such as the Covid-19 pandemic. They emphasis a combination of factors including: making scientific evidence accessible, appropriate and understandable for the political context; improving trust and overcoming misinformation; and facilitating common platforms where different stakeholders from diverse sectors can work to solve problems together[1].For developing countries with more restricted access to resources and sometimes problematic managerial mechanisms such as corruption and nepotism, a deliberate planned approach to KTE can have unique and constructive achievements and benefits.

Our study also uncovered barriers faced by policy makers to engaging in KTE during the COVID-19 pandemic; echoing other studies. Mahendradhata et al. (2021) found that a lack of knowledge about KTE as a concept and how to apply it limited evidence utilisation. They also found that restricted resources, lack of organizational support, lack of critical appraisal skills, and non-aligned missions between researchers and policymakers were further barriers in applying KTE [21]. All these aforementioned barriers along with the financial limitations and cultural and structural barriers can affect engagement in KTE negatively.

Oliver et al. (2014) in a review article have summarized the main barriers and facilitators of applying evidence by policymakers. They have concluded that timely access to high-quality scientific research along with collaboration and communication with the policymakers can act as the main effective factors in applying evidence by policymakers. Lack of access to evidence, non-clarity, suitability, trustworthiness of the findings, timeliness and costs were named as the main barriers of applying evidence by the policymakers [22]. Hasanpoor et al. (2018) have also referred to some barriers of EIPM among healthcare managers as follows: characteristics of decision makers, decision making environment, research and education system, organizational and team barriers. They have also pointed to internal and external factors, inter-personal and social factors as the main facilitators [7].

Our study has also shown that policymakers are aware of the benefits of EIPM, and that this awareness was heightened during the pandemic. Lancaster et al. (2020) also revealed that the COVID-19 pandemic led to an increased interest in and uptake research in their decision making. They emphasized that the pandemic resulted in the opportunity for the judgement of the policymakers` decisions according to their restrictions [23].

And finally, results of our study have shown that a failure to use and apply evidence by policymakers during COVID-19 pandemic led to negative consequences including public dissatisfaction, ineffective decision making, organizational collapse, frustration, conflicts and failure to change amongst staff, prohibited organizational improvement and growth. In this regard, Ioannidis (2020) has also claimed that lack of applying scientific research by policymakers during pandemic may lead to dissemination of misinformation, exaggerated estimations and inappropriate allocation of the resources. All of these can threaten the scientific reputation of public health, media and health policymakers [2].

On the other hand, according to the present results, applying EIPM by the policymakers, is accompanied by some necessities the same as identifying the knowledge gap, determining the lessons learned, producing sustainable and flexible resources, establishing of a research network and legal and ethical mechanism for access to health data. According to Yang (2020), during pandemic, evidence based management should benefit from different standards the same as scientific quality, relatedness, appropriateness and rationality of the evidence. Furthermore, the capacities of applying evidence should be made inside and outside of the government. Yan also strongly emphasized that in a democratic community, policy making should be based on both evidence and the values [24]. This study tries to bring together a comprehensive view of the experiences and attitudes of health managers and policymakers towards the use of evidence in decision making during the Covid-19 pandemic in Iran. The study is also triangulated the knowledge from academic faculty members in the field of knowledge utilization with those of practitioners working at different levels of management and policy making in the Iranian healthcare system. Our conceptual framework is both a descriptive depiction of the themes that emerged in the Iranian context, but can also serve as an analytical framework for similar studies in developing countries.

This study has some limitations. First some interview had to be conducted via telephone due to COVID-19 restrictions at the time which may limit the interactions during the interview. Second, for the same reason most of policymakers and managers had a very restricted time to allocate for the interviews, hence in-depth discussion on issues were not all possible. Third, as this was a qualitative research study, results should be understood in context before applying lessons to different countries and circumstances.

## Conclusions

Large volumes of evidence are being produced during the Covid-19 pandemic. Applying this evidence should be of central concern for health managers and policymakers. The results of this study show that developing purposive KTE strategies and activating KBs can be among the main strategies to move toward EIPM during the pandemic. At the same time attention to local barriers and facilitators of applying evidence can pave the way for policymakers to better identifying the health system`s problems and preparing rapid response in the pandemic condition.

## Data Availability

The datasets used and/or analyzed during the current study are available from the corresponding author on reasonable request.

## Abbreviations

EIPM: Evidence informed policy making
KB: Knowledge Brokers
KTE: Knowledge Translation Exchange
WHO: World Health Organization
PPE: Personal Protective Equipment
SARS: Severe acute respiratory syndrome
MERS: Middle East Respiratory Syndrome
A/H1N1: Influenza A virus subtype H1N1
